# A Pilot Study of Influence of Endurance Training on the Prooxidative and Antioxidant Status of Women after Breast Cancer

**DOI:** 10.3390/ijerph18062822

**Published:** 2021-03-10

**Authors:** Katarzyna Domaszewska, Arkadiusz Janiak, Tomasz Podgórski, Anna Demuth, Jakub Kryściak, Paweł Perkowski, Urszula Czerniak

**Affiliations:** 1 Department of Physiology and Biochemistry, Poznan University of Physical Education, 61-871 Poznań, Poland; podgorski@awf.poznan.pl (T.P.); j.krysciak@awf.poznan.pl (J.K.); 2 Faculty of Health Sciences, Calisia University, 62-800 Kalisz, Poland; a.janiak@akademiakaliska.edu.pl; 3 Department of Anthropology and Biometry, Poznań University of Physical Education, 61-871 Poznań, Poland; demuth@awf.poznan.pl (A.D.); czerniak@awf.poznan.pl (U.C.); 4 Province Polyclinic Hospital, 62-504 Konin, Poland; paper5@tlen.pl

**Keywords:** FRAP, TBARS, anaerobic threshold, peakVO_2_

## Abstract

The objective of this study was to assess the effect of 8-week long endurance training on the prooxidative–antioxidative status of plasma in women treated for breast cancer. The participants of the study were 12 women after radical mastectomy aged 45 to 56 years (M = 50.6 ± 2.9 years), who had undergone full cancer treatment, on average more than 5 years after the treatment (M = 5.9 ± 0.9 years). Body mass components were measured twice using the method of bioelectric impedance analysis. In order to optimize training loads and to assess the level of exercise tolerance of the participants, the group was subject to an ergospirometric exercise test twice, before (1st) and after (2nd) the completion of the training cycle. The blood was also taken twice for biochemical analyses. Statistically significant differences were noted in the maximum exercise load, the level of which increased in the second test (*p* < 0.05). No change was observed in the level of antioxidative potential, i.e., the content of some variables, ferric reducing ability of plasma (FRAP), urea, total phenolics, thiobarbituric acid reactive substances (TBARS), and in the blood indices of the body’s nutritional status during the project (total protein, albumin. Endurance training caused an increase in exercise tolerance and did not cause an aggravation of oxidative stress in women undergoing breast cancer treatment.

## 1. Introduction

Breast cancer is the world’s most common cancer in women, and it accounts for as many as 25% of the total number of cancer cases in this population [1]. Breast cancer treatment should be carried out by a multidisciplinary team, with the participation of an oncology surgeon, clinical oncologist, and radiation therapist in particular. Basic treatment involves surgery, which has gradually evolved towards a less aggressive form over the recent decades. The decision concerning the choice of the type of breast cancer treatment is made on the basis of the evaluation of the stage of the disease according to the Tumor–Node-Metastasis (TNM) classification of malignant tumors, the biological subtype, the patient’s overall condition, and her preferences (e.g., type of surgical treatment). Adjuvant therapy is a systemic pharmacological treatment: chemotherapy, hormone therapy, and anti-HER2 therapy [2]. Early consequences of surgical treatment are related to temporary immobility and a postoperative wound, which lead to the disturbed function of the respiratory system, limited chest mobility, and weakening of the muscular strength. Circulatory insufficiency manifests itself, among others, in orthostatic intolerance [3]. Chemotherapy and radiotherapy as additional treatments result in an increased amount of free oxygen radicals and a decrease in the concentration of antioxidants in the tissue and serum. In natural conditions, such reactive oxygen species are formed during stress, in the course of diseases, during physical exercise, or as a result of harmful effects on the environment, among other things. Excessive amounts of reactive oxygen species (ROS) in damaged cells are destructive for the neighboring cells and disturb their functions [4]. A long-term inflammation leads to tissue anoxia and the production of even larger amounts of free radicals, which are not oxygen derivatives. A body constantly exposed to the action of free radicals develops a number of enzymatic and nonenzymatic mechanisms to prevent or limit the damage caused by that. The most significant enzymatic mechanisms are superoxide dismutase (SOD), catalase (CAT), glutathione peroxidase (GPX), and glutathione reductase (GR). Nonenzymatic mechanisms include, among other things, antioxidants, such as vitamin C (ascorbic acid), glutathione (GSH), tocopherol, free radical scavengers (adrenaline, bilirubin, uric acid), metal ions, i.e., iron, copper, zinc, manganese, selenium building active enzyme centers as well as ceruloplasmin, transferrin, ferritin [5,6]. Many publications indicate that a higher blood concentration of total phenolics protects the body against many civilization-related diseases (sclerosis, cancers) [7]. Moreover, it has been demonstrated that in patients receiving chemotherapy, both for hematological and solid cancers, leukocytes produce more free oxygen radicals than in healthy people. In patients treated with high-dose chemotherapy, a decrease of plasma concentration of antioxidants, such as vitamin C, alpha-tocopherol, and beta-carotene, was noted [8]. At the same time, a significant decrease in the level of GSH (significant endogenous antioxidant) was noted in patients treated with busulfan, carmustine, and cisplatin [8]. In patients, who were administered daunorubicin and cytarabine for acute myeloid leukemia, an increase in the level of MDA-malondialdehyde and a significant fall in SOD, GPX, and total antioxidant status (TAS), important components of the defense system against free radicals, was noted (second drug). At the moment, the problem of cellular antioxidant potential is not routinely considered in the planning of cancer treatment and during its course. It has, however, an indirect link with cancer patient nutrition. We know that a mixture of vitamins and microelements has strong properties supporting the antioxidant response. It is suspected that the application of this type of therapy during some methods of systemic treatment may weaken the cytotoxic effect of chemotherapy, as the escalation of oxidative stress is mentioned in the mechanism of cytotoxic action of some chemotherapeutics [9]. Studies show a close relation between free radicals, the activity of antioxidative processes and their effectiveness correlating with the effectiveness of applied chemotherapy.

The rehabilitation process following a mastectomy includes, among other things, psychological rehabilitation, physical rehabilitation, i.e., physiotherapy, and socio-professional rehabilitation. The need for rehabilitation exercises during cancer treatment is undisputed today. The objective of kinesitherapy is to restore the full range of motion in the joint on the side of the operated limb and prevent lymphedema. Over recent years, increasing attention is given to the role of physical activity undertaken by women after the completion of cancer treatment [10]. Systematic physical training results in improved fitness and physical capacity of the body increased immunity, e.g., through strengthening the antioxidative status of the body. The objective of the study was the assessment of the effect of an 8-week long endurance training on the prooxidative–antioxidative status of plasma in women treated for breast cancer.

## 2. Materials and Methods

The participants of the study were 12 women after radical mastectomy aged 45 to 56 years (M = 50.6 ± 2.9 years) who had undergone full cancer treatment. All participants were in the second and third stages of the disease before starting the treatment. After the surgery, they were administered chemotherapy based on anti-cyclins and taxoids. The type of treatment scheme and treatment duration depended on the stage of cancer according to the TNM staging system (stage IIB and IIIA). The next stage of treatment was radiotherapy. A dose of 50 Gy was routinely applied in 25 fractions on the whole breast and in 10–25 fractions at the site of tumor resection. Each patient had also completed hormonal treatment with tamoxifen (20 mg once a day) and additional treatment with gonadotropin-releasing hormone agonist depending on the stage, presence of risk of relapse and patient’s age (for example, goserelin 3.6 mg administered subcutaneously every 28 days). On average, women taking part in the program had completed cancer treatment more than 5 years prior to the study (M = 5.9 ± 0.9 years) and did not have health contraindications for undertaking physical activity. The studied group was asked not to take vitamins or diet supplements, which could affect the measured biochemical blood indicators in the period before and during the study. The subjects were also asked not to change their dietary habits for the duration of the project and not to perform any additional physical activity, except for the implementation of the research scheme. The study was conducted according to the Declaration of Helsinki and the National Statement and Human Research Ethics Guidelines. It was also approved by the Institute for Research in Biomedicine (IRB) at the Poznan University of Medical Sciences (10 May 2012; Ethics Approval Number: 535/12). The study was performed between June and October 2012 in an accredited endurance test laboratory of the Poznan University of Physical Education. All of the patients from the sample group gave their written informed consent.

### 2.1. Anthropometrics Measurements

Body mass components were measured twice using the method of bioelectric impedance analysis (BIA) by means of Akern—BIA 101 (Akern Bioresearch, Florence, Italy) body composition analyzer [11]. In order to perform body composition analysis, measurements of two basic somatic properties were used (body height in cm and body weight in kg) to calculate the body mass index (BMI; kg/m^2^). The following body mass components were assessed: the levels of body fat (FAT; kg), lean body mass (LBM; kg), and total body water (TBW; L). All measurements were taken in the morning from 7 am; patients were fasting; after taking the blood, they received the same light meal, and after a break, they proceeded to the exercise test. They were instructed not to consume alcohol for at least 48 h before the test, not to do vigorous physical exercise for at least 12 h before, not to use a sauna for at least 12 h before, and to void the bladder 30 min before the test. Patients with ICDs (defibrillators), pacemakers or metal implants, patients with epilepsy, hemiparesis, with wounds or skin lesions on hands or feet were excluded from the study.

### 2.2. International Physical Activity Questionnaire

The level of physical activity was determined in accordance with the criteria of the international physical activity questionnaire (IPAQ). Women who qualified for the project were characterized by low and moderate levels of physical activity. The level of physical activity was categorized according to the criteria of the international physical activity questionnaire. The following levels were adopted: (1) low—physical activity does not meet the criteria for moderate or intensive activity; (2) moderate—physical activity meets at least one of the following criteria: three or more days with at least 20 min of vigorous physical activity; five or more days with at least 30 min of moderate-intensity activity or walking; five or more days of any combination of physical activity with at least 600 MET-min/week, (3) high—three or more days with intensive physical activity exceeding 1500 MET-min/week or five or more days of any combination of physical exercise (walking, moderate-intensity or vigorous activities) exceeding 1500 MET-min/week [12].

### 2.3. Assessment of Aerobic Fitness (Graded Exercise Test Protocol)

The exercise tests were conducted between 8:00 am and noon in an air-conditioned laboratory, 2 h after consuming a light breakfast. The duration of the physical exercise was 20 min. In order to optimize training loads and to assess the level of exercise tolerance of the participants, the group was subject to the ergospirometric exercise test twice, before (1st) and after (2nd) the completion of the training cycle. The test was performed on a cycle ergometer (Kettler DX1 Pro, Ense, Germany): a three-minute warm-up with a 25 W load, after which the load was increased by 10 W every 90 s (60 revolutions per minute (RPM)). The test was performed until refusal or impossibility to maintain the set pace. The exercise tests were conducted between 8:00 am and noon in an air-conditioned laboratory, 2 h after consuming a light breakfast (one sandwich with butter and cheese; approx. 200 kcal).

### 2.4. Physiological Measurements

Expired gases, minute ventilation (Ve) and heart rate (HR) during graded exercise test (GXT) were monitored continuously with an Oxycon mobile automated system (Viasys Healthcare, Höchberg, Germany). Oxygen intake (VO_2_) and carbon dioxide output (VCO_2_) were measured breath-by-breath and were averaged at 15 s time periods. Before each trial, the system was calibrated according to the manufacturer’s instructions. Ambient conditions, i.e., temperature, humidity and barometric pressure, were recorded by the sensors. In a two-point volume calibration (0.2 and 2 L/s), the flow values were measured automatically at the set measuring points. Gas analyzer calibration was carried out with a standard gas mixture of 5% CO_2_ and 16% O_2_. PeakVO_2_ was defined as the highest 15 s averaged VO_2_ obtained during the last exercise in the test. HRmax (beats/min) was measured as the highest 15 s averaged value in the test. Time to exhaustion (TTE) and maximal work rate (WRmax) were also measured. The individual training load at the level of PPA (anaerobic threshold) was established using the “V-slope” method. To determine ventilatory threshold (VT), the V-slope method was administered using computerized regression analysis of the slopes of the CO_2_ output versus O_2_ uptake plot, which detects the beginning of the excess CO_2_ output generated from the buffering of [H^+^] [13]. The method involves the analysis of the behavior of VCO_2_ as a function of VO_2_ during GXT with a consequent increase in VCO_2_. This resulted in a transition in the relation between the VCO_2_ and VO_2_. The software supplied by Viasys Healthcare was supported by visual inspection by an experienced researcher. The ventilatory equivalent method (VEQ method) was used as a secondary method, and the point when the equivalent for oxygen (VE/VO_2_) raised without a concomitant rise in the equivalent for carbon dioxide (VE/VCO_2_) was detected [14,15,16,17,18,19]. The VT was expressed as work rate (W), heart rate (bpm) and time when the VT was reached.

The entire study protocol and results from the cardiopulmonary exercise testing (CPET) are thoroughly described in our previous paper [20].

### 2.5. Blood Collection, Biochemical Measurements

The blood was taken twice for biochemical analyses -at rest, at the beginning, and after the completion of the training program. Blood samples (10 mL) were taken from a vein in the forearm using an S-Monovette blood collection system (Sarstedt, Nümbrecht, Germany). 9 mL of the blood was left to clot at room temperature, then centrifuged (1500 g, 4 °C, 5 min). The collected samples were frozen and stored at −80 °C until the analysis. Blood cell count tests were performed on fresh blood. Total protein, albumin, and urea concentrations were determined directly after taking and centrifuging the blood on the day of the test. Plasma for determining FRAP, TBARS, and total phenolics concentrations was frozen for two months. Biochemical determinations were performed on a sample defrosted once. The following parameters were determined in whole venous blood (1 mL in microtube EDTA K3; Sarstedt, Germany), using MYTHIC 18 hematological analyzer (PZ CORMAY S.A., Lublin, Poland): hemoglobin concentration, hematocrit value, the total count of erythrocytes, leukocytes, thrombocytes, and relative percentage numbers of granulocytes, lymphocytes, and monocytes. Colorimetric methods were used to determine the concentrations of the total antioxidant capacity of plasma FRAP, reference values: 600–1600 μmol/L [21], the plasma concentration of TBARS, reference values: 1–6 μmol/L) [22] and total phenolics (reference values: 2.8–4.0 g GAE/L) [23]. All reagents used for measurements of the above parameters were obtained from (Sigma-Aldrich Chemie GmbH., Steinheim, Germany). Moreover, total protein, albumin and urea concentrations were assessed using the spectrophotometric methods with commercially available reagent kits: Liquick Cor-TOTAL PROTEIN (cat. no. 2-236, sensitivity 1.0 g/L), Liquick Cor-ALBUMIN (cat. no. 2-238, sensitivity 11.4 g/L) and Liquick Cor-UREA (cat. no. 2-261, sensitivity 0.27 mmol/L) diagnostic sets (PZ CORMAY S.A., Poland), respectively. The samples were read using a Synergy 2 SIAFRT multimode microplate reader (BioTek, Winooski, VT, USA). All pre-training and post-training specimens from each individual were analyzed in the same batch by an experienced technician, who was blinded to the origin of samples.

### 2.6. Training Program

The training program offered to participants was performed on a cycle ergometer for two months, three times a week. In total, 28 training sessions, each, including a 5 min warm-up (50–60% HRmax), 30–45 min of the proper part (at the level of individual PPA), a 5 min warm-down, cycling without a load. In the end, each patient performed stretching and breathing exercises for about 15 min. Exercise loads were determined individually on the basis of the ergospirometric exercise test. Each patient had undergone all designated training sessions, which always took place in the morning. The training program and exercise tests were carried out using the same ergometer. Physical training was supervised by a physiotherapist with a medical background.

### 2.7. Statistical Analyses

The distribution of variables was tested by Shapiro–Wilk test of normality. All the data are presented as mean (standard deviation) and median (interquartile range). The differences between paired, normally distributed variables were tested using the paired t-test and, in the case of asymmetrically distributed variables, by the Wilcoxon test. The relationship between the variables was tested while using Pearson’s correlation. All results were statistically analyzed using Dell Inc. (2016) (Dell Statistica v.13, Tulsa, OK, USA).

## 3. Results

A group of women after a completed breast cancer treatment was examined twice, before and after an 8-week long endurance training, in order to assess its effect on the prooxidative–antioxidative status of plasma. The statistical analysis performed showed consistency with the normal distribution of most of the variables analyzed in the study, with the exception of three parameters (TBARS, neutrophils, monocytes), for which the results of parametric (paired *t-*test) and nonparametric tests (Wilcoxon matched-pairs set) were consistent. Thus, in further analysis, the results of the paired t-test were considered. The women were characterized by an average height of 164.9 cm (Me = 163.5 cm, SD = 2.9 cm, Q1 = 159 cm, Q3 = 171.5 cm). A comparison of somatic parameters (Table 1) measured in the first test and in the second one demonstrated that no significant changes in the body mass or in body composition had occurred. Endurance training resulted in an increase in the peak oxygen uptake, but no statistical significance was achieved (*p* < 0.05).

A comparison of values of selected hematological and biochemical blood parameters measured in the first and in the second test is shown in Table 2. Statistically significant differences were noted in the maximum exercise load, the level of which increased in the second test (*p* < 0.05). A slight increase was noted in the level of antioxidative potential, i.e., the content of some variables (FRAP, urea), with no changes or a slight decrease of other biochemical parameters of the blood (total phenolics, TBARS). No change was observed in the blood indices of the body’s nutritional status during the project (total protein, albumin).

The analysis of changes in hematological blood parameters (Table 2) did not indicate substantial significant changes after the completion of the training program by the participants. An increase in the mean concentration of most of the variables was noted, while no change was noted in lymphocytes and monocytes counts compared to the average starting level.

The analysis of relationships between the studied variables, based on the assessment of the strength and direction of relations described by Pearson’s correlation, showed mutual relations. Peak VO_2_ on both dates of tests showed a high negative correlation coefficient with BMI (1st test r = −0.7152, *p* < 0.05; 2nd test r = −0.6181, *p* < 0.05) and positive correlations with the value of maximum load in the 1st test (r = 0.8503, *p* < 0.05) and 2nd test (r = 0.7923, *p* < 0.05). In the first test TBARS concentration showed correlation with erythrocyte (r = −0.6256, *p* < 0.05), hematocrit (r = −0.6952, *p* < 0.05), TBARS levels (r = −0.7300, *p* < 0.05). FRAP concentration on both dates of tests correlated with the protein and total phenolics blood concentration, 1st test (r = 0.8280, *p* < 0.05) for total phenolics and (r = 0.8155, *p* < 0.05) for proteins, respectively. In 2nd test (r = 0.8293, *p* < 0.05) for total phenolics and (r = 0.8293, *p* < 0.05) for proteins, respectively. Total phenolics levels on each date of tests showed high correlation with protein blood concentration (1st test r = 0.9744, *p* < 0.001 and 2nd test r = 0.9744, *p* < 0.001). Only in the 1st test was there correlation between peakVO_2_ and neutrophils (%) (r = 0.6071, *p* < 0.05) and lymphocytes (%) (r = −0.6717, *p* < 0.05). In the 1st test was correlation between Load _VT_ and LBM (r = 0.8473, *p* < 0.05), HR _VT_ and erythrocytes (r = −0.5867, *p* < 0.05), Load _VT_ and peak Load (r = 0.8420, *p* < 0.05). In 2nd test correlation was found only between Load _VT_ and peak Load (r = 0.8288, *p* < 0.05).

## 4. Discussion

In patients, who have undergone breast cancer treatment, impaired function of the cardiovascular and respiratory systems and weakening fatigue often occur both in the early period and a few months or years after the completion of the treatment. Studies have shown that these negative side effects resulting from the treatment process may be limited by applying moderate individualized physical activity. The oxidative stress arising in the treatment process may be reduced with appropriate pharmacotherapy, diet, but also with physical training. Changes caused by increased production of free radicals may be reversible. However, when the rate of free radical production is higher than the rate of repair mechanisms, significant contraction of mitochondria may occur with damage of mitochondrial crest, scattering of cell elements, and damage of cytoplasmatic membranes. Moreover, free radicals affect the transport of glucose, calcium-dependent ATPase activity of creatine kinase activity. In the studied group of women on each date of testing, biochemical indicators, such as FRAP, TBARS, urea, total proteins, and albumins were within the normal physiological range; only concentration of total phenolics on the 1st and second date of testing was below the reference values and amounted to 2.44 ± 0.09 and 2.43 ± 0.28 g GAE/L, respectively. Supervised individualized endurance training did not result in a significant change in any hematological and biochemical indicators, in spite of the increase in the level of exercise tolerance of the participants.

Physical activity undertaken by healthy persons, as well as persons with a low physical capacity, is of great significance for maintaining their good physical fitness and exercise tolerance. It improves the function of the circulatory and respiratory systems. Optimization of the training process and physical activity close to the anaerobic threshold (AT) are the most effective factors affecting post-training changes in the circulatory and respiratory capacity. In the case of patients after cancer treatment, physical activity contributes to an improved functional capacity, thus limiting negative consequences of the immobility or the treatment process itself [24].

The training program underwent by research participants was individualized and adjusted to the level of their circulatory and respiratory capacity. A supervised 2-month long training on a cycle ergometer resulted in no significant change in the peakVO_2_ (25.74 ± 4.04 vs. 27.00 ± 3.68 mL/kg/min) with only a small decrease in the body weight. An increase in physical fitness is also indicated by obtaining a higher maximum load during the ergospirometric exercise test on the 2nd date of tests (112.50 ± 23.0 vs. 123.33 ± 22.09 W). In comparison to the results of studies on the effectiveness of applying endurance training in the rehabilitation process, the results of our study fully confirm the trends of post-training changes in physical capacity and exercise tolerance described by other authors [25,26,27,28]. A properly planned rehabilitation or sports training leads to adaptive changes in the muscle tissue, improvement of metabolic processes, and structural changes in muscles themselves. An increase in muscle strength and increased immunity of the tissue to fatigue are observed. Radical oxygen species secreted during physical exercise as well as growth and inflammatory factors produced as a result of the damage of muscle fibers take place in this process, which leads to an increase in the number of neutrophils, monocytes, and lymphocytes in the blood as well as inflammation markers in the blood [29,30,31,32]. The study of Rajneesh et al. (2008) indicated that in the blood of women with breast cancer, increased concentration of TBARS and other markers of ROS activity was observed as a result of the disease process [33]. Physical activity, in particular of to high-intensity, may cause a further increase in their concentration. In our study, on the 1st and second date, the concentration of TBARS, which is an indicator of cell membrane damage, did not change statistically significantly, which may be considered beneficial for the studied women. At the same time, we noted no statistically significant change in the total FRAP and compounds, such as total phenolics, proteins, albumins, and urea in the blood on both dates of tests, which indicates the prooxidative-antioxidative balance of the body. This may indicate a good tolerance of training loads by the studied women. No reports have been found in the literature to confirm the significance of physical activity and its effect on the concentration of total phenolics in the blood of women treated for breast cancer. However, there are reports of the anti-cancerous activity of some polyphenolic compounds (e.g., chlorogenic acid, resveratrol) by means of reducing inflammation or stimulation of apoptosis of cancerous cells [34]. Additionally, it was proved that phenolics decrease muscle damage caused by an increase in ROS concentration during physical exercise and in the period of restitution [35]. Hence, the amount of polyphenols in the blood depends on the number of consumed foodstuffs rich in phenolics, their absorption from the alimentary tract, and their use by the body [36]. In order to improve the antioxidant potential of plasma, it would seem beneficial to additionally include polyphenol supplementation in the program of patient care. Studies show that administering antioxidants results in an increase of the antioxidant potential of plasma [37]. To summarize the discussion of the significance of antioxidants and negative effects of free radicals, the theory of hormesis can be quoted, in which insignificant oxidative stress originating in cells, e.g., as a result of physical activity, may be a predictor of the development of beneficial cell changes, contributing to the tolerance of acute oxidative stress [38,39]. Drouin et al. [40] studied the effects of repeated physical exercise on hematological changes in breast cancer patients during the treatment process and after its completion. They noted that physical exercise prevents the fall in the erythrocytes counts during radiotherapy. The analysis of the number of erythrocytes, hematocrit value, and the concentration of hemoglobin in the studied group of women showed their statistically insignificant increase on the 2nd date of tests. The basis of this mechanism may be the change in the rate of erythropoiesis regulated by such factors as, among other things, oxygen deficiency, hormonal and nervous factors.

Margolis et al. [41] showed in their study that women after menopause with a larger amount of leukocytes in the blood have an increased risk of breast, large intestine, endometrial, and lung cancer, as well as higher cancer mortality. The total number of leukocytes and relative percentage of granulocytes, lymphocytes, and monocytes of the studied women were within normal ranges. The change in the number of leukocytes during physical exercise depends on its duration, intensity, and level of fitness of the subjects. The physiological mechanism of change in leukocyte count is caused by many neurohormonal factors, including cortisol, catecholamines, growth hormone, endorphins, sex hormones. Another group of factors intensifying exercise leukocytosis is secreted cytokines (TNF, IL-1, IL-6). Additionally, changes in glutamine, glucose, lipids, and heat shock protein concentrations in the blood contribute to the occurrence and intensifying of the mechanism of exercise-induced leukocytosis. In the initial stage of the exercise, the increase in the number of leukocytes is caused by the action of catecholamines, whereas the later, slower increase is due to the effect of cortisol on the bone marrow. During an intensive physical exercise, spontaneous degranulation of neutrophils occurs, with an increase in their phagocytic properties, thus stimulating neutrophil oxygen burst. Similar to other leukocytes, also NK cells are mobilized to the peripheral circulation, influenced by the physical strain. This is linked to the regulatory effect of catecholamines on their function [42]. If a body is subject to physical work with an intensity exceeding the exercise capacity of the exercising person, leukopenia occurs. The cytotoxic ability of NK lymphocytes is impaired, as is the proliferation and secretion ability of other types of lymphocytes [43]. The training program carried out by women after cancer treatment did not result in a significant change in the number of leukocytes and in their percentage.

## 5. Conclusions

In the group of patients after breast cancer treatment, endurance training caused an increase in exercise tolerance and did not cause aggravation of oxidative stress in women undergoing breast cancer treatment.

Limitation of the Study

The study was carried out on a specially selected group. The group was fully representative of a population of women who met the following conditions: they had undergone a radical mastectomy in the second and third stages of the disease and received full oncological treatment: chemotherapy, radiotherapy, and hormone therapy. For this reason, it is difficult to project the results for the whole population of women after cancer treatment. The limitation of the study is the small number of patients, the lack of a control group and the inability to compare results with the effects of endurance training in women at various times after the completed oncological treatment.

## Figures and Tables

**Table 1:** Somatic and physical capacity parameters in sessions 1 and 2.

Parameter	1st Date (*n* = 12) Mean ± SD Me ± Q_1_–Q_3_	2nd Date (*n* = 12) Mean ± SD Me ± Q_1_–Q_3_	*p*-Value
Body mass (kg)	66.50 ± 8.12 65.0 ± 59.5–73.0	65.75 ± 8.18 65.5 ± 61.5–69.5	0.3568
BMI (kg/m^2^)	24.45 ± 2.27 24.8 ± 23.2–25.3	24.28 ± 2.28 25.5 ± 23.6–25.2	0.4834
LBM (kg)	45.32 ± 3.78 45.5 ± 43.2–47.7	44.67 ± 3.88 45.9 ± 42.2–47.6	0.1992
TBW (L)	33.18 ± 2.76 33.4 ± 31.6–34.9	32.68 ± 2.84 33.6 ± 30.9–34.8	0.1808
FAT (kg)	21.25 ± 5.22 19.0 ± 17.9–25.1	21.28 ± 5.50 19.5 ± 18.8–22.8	0.9722
_VT_ HR (beat/min)	127.75 ± 13.07 124.0 ± 119.0–139.0	142.25 ±13.06 144.0 ± 138.0–148.0	<0.0001
_VT_ Load (W)	76.67 ± 13.37 75.0 ± 75.0–85.0	94.17 ± 14.29 90.0 ± 85.0–105.0	0.011
peak HR (beat/min)	158.92 ± 15.37 162.0 ± 144.0–171.5	166.50 ± 13.56 170.0 ± 162.5–175.5	0.2134
peakVO_2_ (mL/kg/ min)	25.74 ± 4.04 24.4 ± 22.9–28.7	27.00 ± 3.68 26.3 ± 23.4–30.6	0.1658
peak Load (W)	112.50 ± 23.01 105.0 ± 100.0–130.0	123.33 ± 22.09 120.0 ± 105.0–140.0	0.0155

Data are expressed as mean ± SD and Me ± Q1-Q3, * *p* ≤ 0.05 vs. II session; abbreviations: BMI = body mass index, LBM = lean body mass, TBW = total body water, FAT = fat body mass, _VT_ HR = heart rate on VT, _VT_ Load = load on VT, peak HR = peak heart rate, peakVO_2_ = peak oxygen uptake.

**Table 2:** Values of selected hematological and biochemical blood parameters in sessions 1 and 2.

Parameter	1st Date (*n* = 12) Mean ± SD Me ± Q_1_-Q_3_	2nd Date (*n* = 12) Mean ± SD Me ± Q_1_-Q_3_	*p-*Value
Erythrocytes (10^12^/L)	4.50 ± 0.40 4.6 ± 4.3–4.8	4.67 ± 0.31 4.7 ± 4.5–4.9	0.0620
Hematocrit (%)	41.1±3.0 40.7 ± 40.3–42.3	42.1 ± 2.1 42.4 ± 40.4–43.5	0.1402
Hemoglobin (mmol/L)	8.89 ± 0.75 8.7 ± 8.6–9.1	9.02 ± 0.44 9.1 ± 8.7–9.4	0.4573
Leukocytes (10^9^/L)	5.32 ± 1.37 5.5 ± 4.8–6.0	5.89 ± 1.53 5.6 ± 4.8–7.1	0.0765
Neutrophils (%)	58.34 ± 10.43 60.6 ± 52.7–63.2	62.27 ± 10.90 65.6 ± 56.9–67.8	0.0843
Lymphocytes (%)	36.13 ± 10.36 32.8 ± 31.7–40.3	32.70 ± 10.12 30.1 ± 27.1–38.9	0.1095
Monocytes (%)	5.53 ± 1.41 5.2 ± 4.9–5.6	5.03 ± 1.15 4.8 ± 4.3–5.4	0.2132
Total proteins (g/L)	68.30 ± 9.46 68.4 ± 60.4–75.9	72.95 ± 14.02 73.7 ± 61.8–85.4	0.2593
Albumins (g/L)	37.62 ± 3.90 36.7 ± 34.4–40.9	39.79 ± 7.29 38.9 ± 35.9–44.9	0.1857
Total phenolics (g GAE/L)	2.44 ± 0.09 2.4 ± 2.4–2.5	2.43 ± 0.28 2.4 ± 2.3–2.7	0.9199
FRAP (μmol/L)	857.25 ± 147.17 873.8 ± 750.4–945.6	859.67 ± 148.65 846.5 ± 750.0–973.2	0.9554
TBARS (μmol/L)	5.09 ± 2.09 4.8 ± 4.1–5.3	5.02 ± 1.81 5.0 ± 3.9–5.4	0.6949
Urea (mmol/L)	3.32 ± 2.09 3.4 ± 2.6–4.0	3.71 ± 1.99 3.9 ± 3.0–4.7	0.5427

Data are expressed as mean ± SD and Me ± Q1-Q3, * *p* ≤ 0.05 vs. II session; abbreviations: FRAP = ferric reducing ability of plasma, TBARS = thiobarbituric acid reactive substances.

## Data Availability

The data presented in this study are available on request from the corresponding author. The data are not publicly available due to the consent provided by participants on the use of confidential data.

## References

[1] Domaszewska K., Pieńkowski T., Janiak A., Bukowska D., Laurentowska M. (2019). The influence of soft tissue therapy on respiratory efficiency and chest mobility of women suffering from breast cancer. Int. J. Environ. Res. Public Health..

[2] Mendes D., Alves C., Afonso N., Cardoso F., Passos-Coelho J.L., Costa L., Andrade S., Batel-Marques F. (2015). The benefit of HER2-targeted therapies on overall survival of patients with metastatic HER2-positive breast cancer–a systematic review. Breast Cancer Res..

[3] Marianne E., Jensen A.B. (2011). Late effects of breast cancer treatment and potentials for rehabilitation. Acta Oncol..

[4] Halliwell B. (1994). Free radicals, antioxidants, and human disease: Curiosity, cause, or consequence?. Lancet.

[5] Halliwell B. (1991). Reactive oxygen species in living systems: Source, biochemistry, and role in human disease. Am. J. Med..

[6] Reilly P.M., Schiller H.J., Bulkley G.B. (1991). Pharmacologic approach to tissue injury mediated by free radicals and other reactive oxygen metabolites. Am. J. Surg..

[7] Hertog M.G., Kromhout D., Aravanis C., Blackburn H., Bbuzina R., Fidanza F., Giampaoli S., Jansen A., Menotti A., Nedeljkovic S. (1995). Flavonoid intake and long-term risk of coronary heart disease and cancer in the seven countries study. Arch. Intern. Med..

[8] Jonas C.R., Puckett A.B., Jones D.P., Griffith D.P., Szeszycki E.E., Bergman G.F., Furr C.E., Tyre C., Carlson J.L., Galloway J.R. (2000). Plasma antioxidant status after high-dose chemotherapy: A randomized trial of parenteral nutrition in bone marrow transplantation patients. Am. J. Clin. Nutr..

[9] Goroshinskaya I.A., Kit O.I., Zuderman N., Ushakova N.D., Lysenko I.B., Nemashkalova L.A., Nikolaeva N.V., Kapuza E.A., Shatokhina O.N. (2018). Oxidative processes in the blood during chemotherapy and lethality of patients with multiple myeloma. J. Clin. Oncol..

[10] McNeely M.L., Campbell K.L., Rowe B.H., Klassen T.P., Mackey J.R., Courneya K.S. (2006). Effects of exercise on breast cancer patients and survivors: A systematic review and meta-analysis. Can. Med. Assoc. J..

[11] Lukaski H.C., Johnson P.E., Bolonchuk W.W., Lykken G.I. (1985). Assessment of fat-free mass using bioelectrical impedance measurements of the human body. Am. J. Clin. Nutr..

[12] Biernat E., Stupnicki R., Lebiedziński B., Janczewska L. (2008). Assessment of physical activity by applying IPAQ questionnaire. J. Phys. Educ. Sport..

[13] Beaver W.L., Wasserman K., Whipp B.J. (1986). A new method for detecting anaerobic threshold by gas exchange. J. Appl. Physiol..

[14] Davis J.A., Whipp B.J., Wasserman K. (1980). The relation of ventilation to metabolic rate during moderate exercise in man. Eur. J. Appl. Physiol. Occup. Physiol..

[15] Gitt A.K., Winter U.J., Fritsch. J., Pothoff G., Sedlak M., Ehmanns S., Ostmann H., Hilger H.H. (1994). Comparison of four different methods for respiratory determination of the anaerobic threshold in normal people, and heart-and lung patients. Z. Kardiol..

[16] Powers S.K., Dodd S., Garner R. (1984). Precision of ventilatory and gas exchange alterations as a predictor of the anaerobic threshold. Eur. J. Appl. Physiol. Occup. Physiol..

[17] Reinhard U., Müller P.H., Schmülling R.M. (1979). Determination of anaerobic threshold by the ventilation equivalent in normal individuals. Respiration.

[18] Shimizu M., Myers J., Buchanan N., Walsh D., Kraemer M., McAuley P., Froelicher V.F. (1991). The ventilatory threshold: Method, protocol, and evaluator agreement. Am. Heart J..

[19] Day J.R., Rossiter H.B., Coats E.M., Skasick A., Whipp B.J. (2003). The maximally attainable VO_2_ during exercise in humans: The peak vs. maximum issue. J. Appl. Physiol..

[20] Kryściak J., Domaszewska K., Czerniak U. (2017). Influence of 12-Week Endurance Training on the Physical Efficiency of Women after Mastectomy.

[21] Benzie I.F., Strain J.J. (1996). The ferric reducing ability of plasma (FRAP) as a measure of “antioxidant power”: The FRAP assay. Anal. Biochem..

[22] Ohkawa H., Ohishi N., Yagi K. (1979). Assay for lipid peroxides in animal tissues by thiobarbituric acid reaction. Anal. Biochem..

[23] Singleton V.L., Rossi J.A. (1965). Colorimetry of total phenolics with phosphomolybdic-phosphotungstic acid reagents. Am. J. Enol. Vitic..

[24] Kindermann W., Simon G., Keul J. (1979). The significance of the aerobic-anaerobic transition for the determination of work load intensities during endurance training. Eur. J. Appl. Physiol..

[25] Neilson H.K., Farris M.S., Stone C.R., Vaska M.M., Brenner D.R., Friedenreich C.M. (2017). Moderate-vigorous recreational physical activity and breast cancer risk, stratified by menopause status: A systematic review and meta-analysis. Menopause.

[26] Ekelund U., Steene-Johannessen J., Brown W.J., Fagerland M.W., Owen N., Powell K.E., Bauman A., Lee I.M. (2016). Lancet Physical Activity Series 2 Executive Committee; Lancet Sedentary Behavior Working Group. Does physical activity attenuate, or even eliminate, the detrimental association of sitting time with mortality? A harmonised meta-analysis of data from more than 1 million men and women. Lancet.

[27] Egan B., Zierath J.R. (2013). Exercise metabolism and the molecular regulation of skeletal muscle adaptation. Cell Metabolism..

[28] Kirkham A.A., Campbell K.L., McKenzie D.C. (2013). Comparison of aerobic exercise intensity prescription methods in breast cancer. Med. Sci. Sports Exerc..

[29] Steinbacher P., Eckl P. (2015). Impact of oxidative stress on exercising skeletal muscle. Biomolecules.

[30] Yang W., Hu P. (2018). Skeletal muscle regeneration is modulated by inflammation. J. Orthop. Transl..

[31] Sass F.A., Fuchs M., Pumberger M., Geissler S., Duda G.N., Perka C., Schmidt-Bleek K. (2018). Immunology guides skeletal muscle regeneration. Int. J. Mol. Sci..

[32] Pedersen B.K., Hoffman-Goetz L. (2000). Exercise and the immune system: Regulation, integration, and adaptation. Physiol. Rev..

[33] Rajneesh C.P., Manimaran A., Sasikala K.R., Adaikappan P. (2008). Lipid peroxidation and antioxidant status in patients with breast cancer. Singapore Med. J..

[34] Paszkiewicz M., Budzyńska A., Różalska B. (2012). Immuno-modulating role of plant polyphenols. Postepy Hig. Med. Dosw..

[35] Malaguti M., Angeloni C., Hrelia S. (2013). Polyphenols in exercise performance and prevention of exercise-induced muscle damage. OMCL.

[36] Scalbert A., Williamson G. (2000). Dietary intake and bioavailability of polyphenols. J. Nutr..

[37] Partyka L., Hartwich J., Dróżdż W., Gruca A., Jopek R. (2001). Changes in oxidative stress parameters in patients with peripheral vascular disease in response to conservative and surgical treatment. Acta Angiol..

[38] Radak Z., Chung H.Y., Goto S. (2005). Exercise and hormesis: Oxidative stress-related adaptation for successful aging. Biogerontology..

[39] Radak Z., Naito H., Kaneko T., Tahara S., Nakamoto H., Takahashi R., Pelaez F.C., Goto S. (2002). Exercise training decreases DNA damage and increases DNA repair and resistance against oxidative stress of proteins in aged rat skeletal muscle. Pflugers Arch..

[40] Drouin J.S., Young T.J., Beeler J., Byrne K., Birk T.J., Hryniuk W.M., Hryniuk L.E. (2006). Random control clinical trial on the effects of aerobic exercise training on erythrocyte levels during radiation treatment for breast cancer. Cancer.

[41] Margolis K.L., Rodabough R.J., Thomson C.A., Lopez A.M., McTiernan A. (2007). Prospective study of leukocyte count as a predictor of incident breast, colorectal, endometrial, and lung cancer and mortality in postmenopausal women. Arch. Intern. Med..

[42] Timmons B.W., Cieslak T. (2008). Human natural killer cell subsets and acute exercise: A brief review. Exerc. Immunol. Rev..

[43] Timmons B.W., Tarnopolsky M.A., Bar-Or O. (2004). Immune responses to strenuous exercise and carbohydrate intake in boys and men. Pediatr. Res..

